# Cancer Patients’ Willingness to Take COVID-19 Vaccination: A Nationwide Multicenter Survey in Korea

**DOI:** 10.3390/cancers13153883

**Published:** 2021-08-01

**Authors:** June Young Chun, Se Ik Kim, Eun Young Park, Sang-Yoon Park, Su-Jin Koh, Yongjun Cha, Heon Jong Yoo, Jae Young Joung, Hong Man Yoon, Bang Wool Eom, Chul Min Park, Ji-Youn Han, Miso Kim, Dae-Won Lee, Jae-Weon Kim, Bhumsuk Keam, Maria Lee, Tae Min Kim, Young Ju Choi, Yoon Jung Chang, Myong Cheol Lim

**Affiliations:** 1 Division of Infectious Disease, Department of Internal Medicine, National Cancer Center, Goyang 10408, Korea; june.y.chun@ncc.re.kr (J.Y.C.); yjc@ncc.re.kr (Y.J.C.); 2 Department of Obstetrics and Gynecology, Seoul National University College of Medicine, Seoul 03080, Korea; seikkim1@snu.ac.kr (S.I.K.); kjwksh@snu.ac.kr (J.-W.K.); marialee@snu.ac.kr (M.L.); 3 Biostatistics Collaboration Team, Research Core Center, National Cancer Center, Goyang 10408, Korea; 13140@ncc.re.kr; 4 Department of Statistics and Data Science, Yonsei University, Seoul 03722, Korea; 5 Center for Gynecologic Cancer, National Cancer Center, Goyang 10408, Korea; parksang@ncc.re.kr; 6 Department of Hematology and Oncology, Ulsan University Hospital, Ulsan University College of Medicine, Ulsan 44033, Korea; sujinkoh@uuh.ulsan.kr; 7 Center for Colon Cancer, National Cancer Center, Goyang 10408, Korea; yongjuncha@ncc.re.kr; 8 Department of Obstetrics and Gynecology, Chungnam National University Sejong Hospital, Chungnam National University School of Medicine, Daejeon 30099, Korea; bell4184@cnuh.co.kr; 9 Center for Urologic Cancer, National Cancer Center, Goyang 10408, Korea; urojy@ncc.re.kr; 10 Center for Gastric Cancer, National Cancer Center, Goyang 10408, Korea; red10000@ncc.re.kr (H.M.Y.); kneeling79@ncc.re.kr (B.W.E.); 11 Department of Obstetrics and Gynecology, Jeju National University Hospital, Jeju 63241, Korea; obgymd@jejunu.ac.kr; 12 Center for Lung Cancer, National Cancer Center, Goyang 10408, Korea; jymama@ncc.re.kr; 13 Department of Internal Medicine, Seoul National University Hospital, Seoul 03080, Korea; misokim@snu.ac.kr (M.K.); daewonlee@snu.ac.kr (D.-W.L.); bhumsuk@snu.ac.kr (B.K.); gabriel9@snu.ac.kr (T.M.K.); 14 Department of Family Medicine, National Cancer Center, Goyang 10408, Korea; eunicemd@ncc.re.kr; 15 Department of Cancer Control and Population Health, National Cancer Center Graduate School of Cancer Science and Policy, National Cancer Center, Goyang 10408, Korea; 16 Center for Clinical Trials, National Cancer Center, Goyang 10408, Korea; 17 Division of Tumor Immunology, National Cancer Center, Goyang 10408, Korea

**Keywords:** cancer, COVID-19, SARS-CoV-2, vaccine, hesitancy

## Abstract

**Simple Summary:**

Despite the importance of vaccination against Coronavirus disease 2019 (COVID-19) in cancer patients, general vaccine uptake rates among cancer patients are known to be low. Here, we tried to investigate the attitude and acceptance rates for COVID-19 vaccine in cancer patients and identify predictive factors for vaccination that could be modified to promote vaccine uptake rates. Between February and April 2021, a total of 1001 cancer patients from five institutions participated in a paper-based survey, consisting of 58 items over six domains. Among the respondents, 61.8% were willing to receive the COVID-19 vaccine. Along with the previously reported predictors for COVID-19 vaccination, including male gender, older age, and influenza vaccination history, we distinctively found that patient’s disease status and health status (absence of cancer recurrence, time since cancer diagnosis over 5 years, and higher EuroQol Visual Analogue Scale scores) were associated with higher acceptance rates of vaccination. Furthermore, physician’s recommendations effectively reduced patient’s vaccine hesitancy.

**Abstract:**

Considering the high morbidity and mortality of Coronavirus disease 2019 (COVID-19) in patients with malignancy, they are regarded as a priority for COVID-19 vaccination. However, general vaccine uptake rates among cancer patients are known to be lower than in their healthy counterparts. Thus, we aimed to investigate the attitude and acceptance rates for the COVID-19 vaccine in cancer patients and identify predictive factors for vaccination that could be modified to increase vaccine uptake rates, via a paper-based survey (58 items over six domains). A total of 1001 cancer patients participated in this nationwide, multicenter survey between February and April 2021. We observed that 61.8% of respondents were willing to receive the COVID-19 vaccine. Positive predictive factors found to be independently associated with vaccination were male gender, older age, obesity, previous influenza vaccination history, absence of cancer recurrence, time since cancer diagnosis over 5 years, and higher EuroQol Visual Analogue Scale scores. Along with the well-known factors that are positively correlated with vaccination, here, we report that patients’ disease status and current health status were also associated with their acceptance of the COVID-19 vaccination. Moreover, 91.2% of cancer patients were willing to be vaccinated if their attending physicians recommend it, indicating that almost 30% could change their decision upon physicians’ recommendation. Unlike other factors, which are unmodifiable, physicians’ recommendation is the single modifiable factor that could change patients’ behavior. In conclusion, we firstly report that Korean cancer patients’ acceptance rate of the COVID-19 vaccination was 61.8% and associated with disease status and current health status. Physicians should play a major role in aiding cancer patients’ decision-making concerning COVID-19 vaccines.

## 1. Introduction

An unprecedented outbreak of the severe acute respiratory syndrome coronavirus 2 (SARS-CoV-2) resulted in more than 163 million people (2% of the global population) being diagnosed with the Coronavirus disease 2019 (COVID-19) and 3 million deaths worldwide [1]. In Korea, 132,818 people had been diagnosed with COVID-19 by 18 May 2021, since the first diagnosis on 20 January 2020 [2]. Clinically, COVID-19 exhibits a variety of manifestations, ranging from asymptomatic to severe pneumonia with multi-organ dysfunction [3]. Individuals with multiple comorbidities and immunocompromised status are known to have worse clinical outcomes and increased mortality from COVID-19 [4,5].

Cancer is a leading cause of death worldwide, accounting for 19 million new cases and 10 million deaths annually [6,7]. The COVID-19 pandemic has disrupted the optimal management of cancer patients: not only scheduled treatment, but also visits to outpatient clinics and hospitalisations, have been interrupted. At the same time, cancer patients are at a high risk of developing severe COVID-19, particularly those undergoing active cancer treatment or with metastatic disease [8,9,10,11]. As cancer patients tend to be of an advanced age and have comorbidities, special attention must be paid to them [12].

Accordingly, the European Society of Medical Oncology [13], American Society of Clinical Oncology [14], and National Comprehensive Cancer Network [15] have all recommended COVID-19 vaccines for cancer patients with a high priority, unless they have contraindications to vaccination. Nevertheless, evidence on vaccine immunogenicity and the durability of vaccine protection is still insufficient in cancer patients, especially those who are immunocompromised or undergoing specific cancer treatment [13,14,15].

For the purpose of pandemic control, in addition to safe and efficacious vaccines against SARS-CoV-2, a high rate of vaccination coverage is essential. However, some might have a sceptical and negative view of vaccines [16,17]. Generally, influenza vaccination rates among cancer patients are known to be low because of concerns about interaction between the vaccine and the malignant disease, as well as the potential side effects [18,19]. Such vaccine hesitancy might hinder the rapid and wide dissemination of COVID-19 vaccination. Nevertheless, limited data are available on the acceptance rate of COVID-19 vaccines in cancer patients. In the literature, researchers have investigated the attitudes toward COVID-19 vaccines in French, Polish, and Romanian cancer patients [20,21,22]. However, none of them presented vaccine acceptance rates according to the primary cancer types or investigated the relationship between the acceptance rate and patients’ experience during the COVID-19 pandemic, disease status, and current health status, which might be strongly associated with the vaccine hesitancy.

Thus, we conducted a nationwide, multicenter survey to examine willingness to take the COVID-19 vaccination and comprehensively identify factors associated with the acceptance rate among patients with various malignancies in Korea. Initially, we predicted that cancer patients’ vaccine hesitancy would be considerable and assumed physicians’ recommendations could reduce this reluctance. To ascertain this, we also investigated how cancer patients’ acceptance rate of COVID-19 vaccination changes according to physicians’ recommendations.

## 2. Methods

### 2.1. Study Population and Questionnaire

We included Korean cancer patients from five institutions (all located in cities) based on the following conditions: (1) aged ≥20 years; (2) visited outpatient clinics between February and April 2021; (3) agreed to participate in this study and provided informed consent. Patients were excluded if they: (1) received any type of COVID-19 vaccine; (2) refused to participate in the survey; (3) had difficulty reading and understanding Korean.

All patients were asked to complete questionnaires consisting of 58 items over six domains: (1) demographic information (10 items); (2) personal experience during COVID-19 pandemic (15 items); (3) past experience related to vaccinations, such as influenza vaccines (9 items); (4) cancer diagnosis and treatment (8 items); (5) current health status, measured by the Korean version of the EuroQol-5 Dimension 3-level questionnaire (EQ-5D-3L; 5 items) and a visual analogue scale (VAS) [23]; (6) attitudes toward COVID-19 and COVID-19 vaccines and willingness to take the vaccination (10 items). The English version of the questionnaire is presented in the Supplementary Material.

The primary outcome of the current study was cancer patients’ acceptance rate of the COVID-19 vaccination. In response to the question on intention to receive the COVID-19 vaccination, each patient chose one of the following responses: ‘willingness to be vaccinated,’ ‘refusal,’ and ‘unsure how to respond’.

### 2.2. Statistical Analysis

For the analysis, if an individual was diagnosed with multiple cancers, each was counted as a separate case. The cancer treatments that patients received were classified into surgery, radiation, chemotherapy, targeted therapy, hormone therapy, immunotherapy, and their combinations. If patients were initially diagnosed with any type of malignancies more than five years before the date of the survey, they were regarded as cancer survivors ≥5 years.

Based on the self-reported height and body weight, we calculated each patient’s body mass index (BMI), and classified the patients into four BMI categories according to the WHO cut-offs for the Asian population [24]: <18.5 kg/m^2^ (underweight), 18.5–22.9 kg/m^2^ (normal), 23.0–24.9 kg/m^2^ (overweight), and ≥25.0 kg/m^2^ (obese). The EQ-5D-3L health states were converted into index values to calculate an individual’s health score.

In addition to descriptive analyses, differences in characteristics were also evaluated among the three groups. We used the analysis of variance or Kruskal–Wallis test for continuous variables, and the Pearson’s chi-squared test or Fisher’s exact test for categorical variables. The logistic regression model was used to investigate the effect of each factor on the acceptance of COVID-19 vaccination. In multivariable analyses, we specifically used sex-stratified conditional logistic model, as this study skewed the general demographic variable, gender, due to the many gynecologic cancers. This model is appropriate when the stratum effect is not of interest and contains sex-specific intercepts, but is conditioned out of the model along with the nuisance parameter. The variables with univariable *p*-value < 0.2 were included in the model and the final model was determined using backward selection with an elimination criterion of *p*-value > 0.05. The results were presented with odds ratio (OR) with 95% confidence intervals (CIs), and a reported *p*-value < 0.05 was considered statistically significant. All statistical analyses were performed using the SAS version 9.4 (SAS Institute Inc., Cary, NC, USA) and R project software (version 3.6.2).

## 3. Results

**Figure 1:**
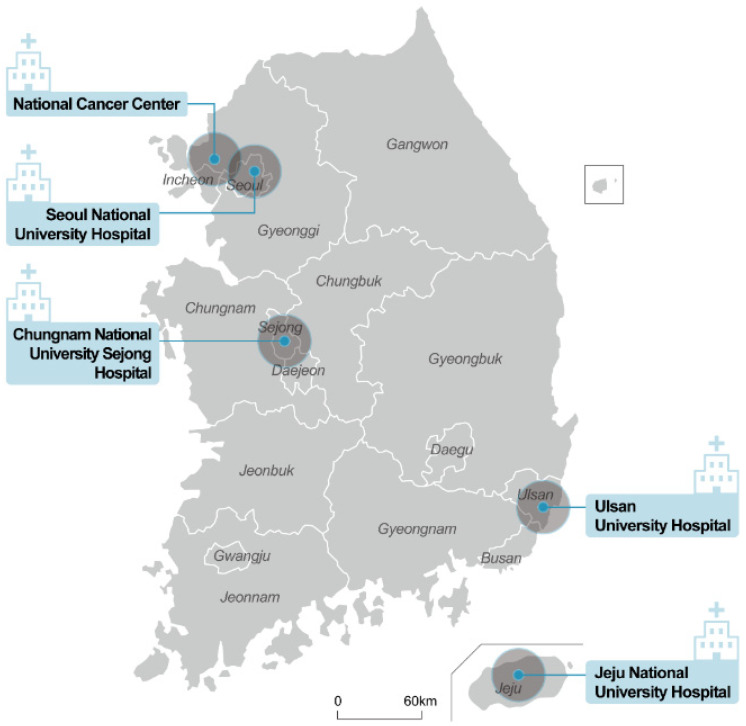
Geographic distribution of the five institutions participated in this study.

**Figure 2:**
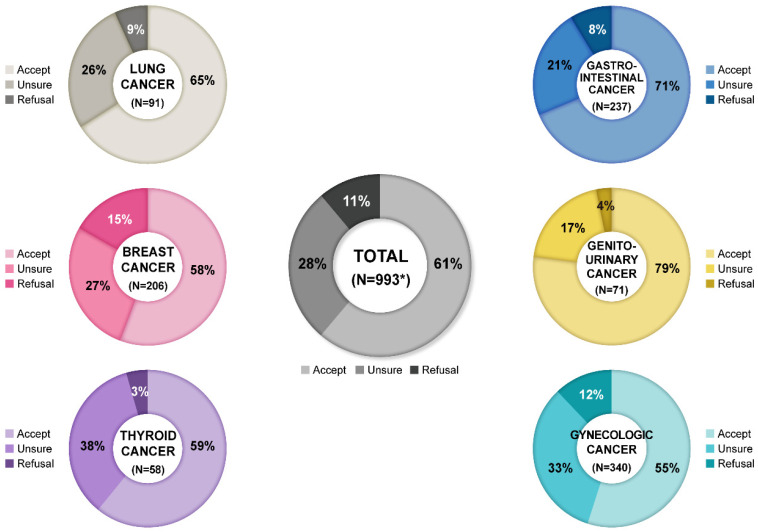
Patients’ intention to take COVID-19 vaccination according to the major malignancy types. * Total number is not the sum of cancer types, since duplication is allowed.

A total of 1001 cancer patients from five institutions participated in the survey. Figure 1 shows the geographic distribution of the five institutions in Korea. Eight patients did not respond to the question regarding intention to take COVID-19 vaccination. Among the respondents, 608 (61.2%) reported willingness to take COVID-19 vaccination, whereas 108 (10.9%) expressed their refusal. The other 277 (27.9%) were unsure how to respond. Figure 2 depicts patients’ intention to receive COVID-19 vaccination according to the major malignancy types.

Table 1 presents patients’ characteristics. The mean patient age was 57.4 years, and 71.7% were female. More than one-fourth (27.0%) were employed, 61.8% were living in the Seoul metropolitan area, and 62.7% had a high school diploma or less. While 68.4% of the patients were non-smokers, 4.3% were smokers at the time of the survey. Hypertension was the most common comorbidity (24.1%), followed by dyslipidemia (10.7%) and diabetes (10.2%), and 17.2% of patients had multiple comorbidities. Moreover, 89.4% had single primary site cancer, while 8.6% and 2.0% had two and more than two primary sites, respectively.

Table 2 provides detailed information on cancer diagnosis and the treatment of all cancer patients and the intention to receive COVID-19 vaccination. Chemotherapy (68.0%) was the most commonly administered treatment modality, followed by surgery (66.4%) and radiation (26.8%). Immunotherapy was conducted in 9.9% of patients. At the time of the survey, 26.8% had recurrent disease, and 73.3% were on cancer treatment.

The results of EQ-5D-3L are presented in Table S1. While 91.3%, 77.8%, and 77.2% of patients did not have difficulties in self-care, mobility, and usual activities, respectively, approximately half (46.0%) and a third (37.6%) complained of pain/discomfort and anxiety/depression, respectively. Mean values of EQ-5D-3L health score and VAS were 0.9 and 72.2, respectively.

In terms of personal experience during the COVID-19 pandemic, 55.6% of cancer patients had taken the COVID-19 test, and 0.3% had been diagnosed with COVID-19 (Table S2). Scheduled outpatient clinic visits and hospital admissions were delayed in 4.3% and 2.8% of the patients, respectively. Meanwhile, 2.6% and 2.4% reported that they could not receive and/or changed planned treatment and testing, respectively.

Further analyses were conducted after dividing patients into two groups: acceptance (willingness to be vaccinated: *n* = 608) and non-acceptance (refusal or unsure of being vaccinated: *n* = 385) groups. In the acceptance group, the most common reason for acceptance was that they trusted the COVID-19 vaccine’s efficacy (46.7%), followed by the existence of an underlying malignancy (30.4%) and trust in the safety of COVID-19 vaccines (11.4%) (Table 3).

In the non-acceptance group, the most common reason was that they were concerned about the COVID-19 vaccine’s safety (41.8%), followed by mistrust in the efficacy of COVID-19 vaccines (20.5%) and lack of information about COVID-19 vaccines (15.3%). Approximately 10% of the non-acceptance group were hesitant or against COVID-19 vaccination owing to underlying malignancies (Table 3).

**Figure 3:**
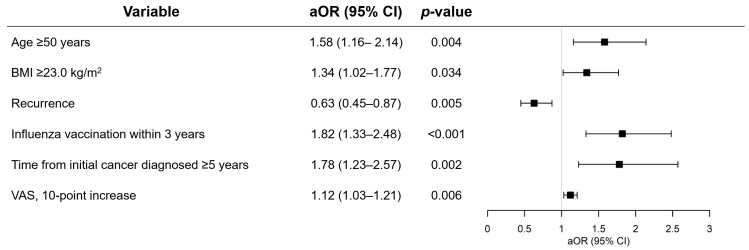
Results of multivariable logistic regression analysis for COVID-19 vaccination acceptance.

Next, we investigated factors associated with the acceptance of COVID-19 vaccination (Table 4 and Figure 3). Overall, female cancer patients, compared to male cancer patients, were reluctant to accept COVID-19 vaccination, as revealed in both univariable (OR, 0.38; 95% CI, 0.28–0.51; *p* < 0.001) and multivariable analyses, adjusting for confounders (aOR, 0.40; 95% CI, 0.29–0.56; *p* < 0.001). In the sex-stratified univariable analyses, a 0.1-point increase in the EQ-5D-3L health score (OR, 1.12; 95% CI, 1.01–1.26; *p* = 0.041) and 10-point increase in the VAS (OR, 1.12; 95% CI, 1.04–1.21; *p* = 0.003) were associated with higher acceptance rates of COVID-19 vaccination. In the sex-stratified multivariable analyses, age ≥ 50 years (aOR, 1.58; 95% CI, 1.16–2.14; *p* = 0.004), BMI ≥ 23.0 kg/m^2^ (aOR, 1.34; 95% CI, 1.02–1.77; *p* = 0.034), influenza vaccination within the last 3 years (aOR, 1.82; 95% CI, 1.33–2.48; *p* < 0.001), and cancer survivors ≥ 5 years (aOR, 1.78; 95% CI, 1.23–2.57; *p* = 0.002), and 10-point increase in the VAS (aOR, 1.12; 95% CI, 1.03–1.21; *p* = 0.006) were associated with increased willingness to be vaccinated (Figure 3). In contrast, experience of cancer recurrence was associated with a decreased willingness to be vaccinated (aOR, 0.63; 95% CI, 0.45–0.87; *p* = 0.005). Education, smoking, primary site of malignancy, current cancer treatment, and the administration of immunotherapy did not affect cancer patients’ acceptance rate of the vaccine (Figure 3).

**Figure 4:**
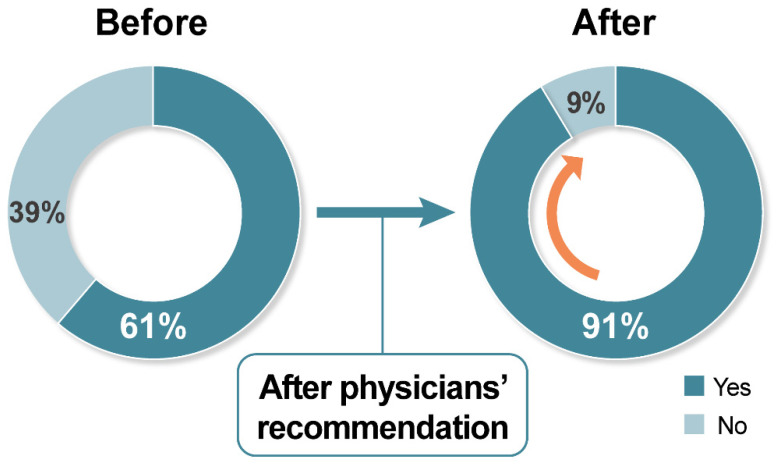
Changes in cancer patients’ acceptance rate for COVID-19 vaccination after physicians’ recommendations.

Lastly, we investigated changes in cancer patients’ intention to receive COVID-19 vaccination when their physicians recommended taking the COVID-19 vaccination. Among the 990 patients who responded to this question, a significant increase in acceptance rate, from 61.2% (baseline) to 91.3% (post-recommendation), was observed (McNemar test *p* < 0.001) (Figure 4).

## 4. Discussion

In this multicenter survey study of Korean cancer patients, 61.8% of the respondents indicated their willingness to take the COVID-19 vaccination, which is higher than that in the French survey, including 999 cancer patients (53.7%) [20], and similar to that in the Polish survey, including 635 cancer patients (60.3%) [21]. Considering the differences in survey timing (French study took place between November and December 2020; Polish study took place between January and February 2021; our study took place between February and April 2021), accumulated global evidence of the COVID-19 vaccine’s efficacy and safety might have been an influencing factor on Korean cancer patients’ intentions.

Whenever humanity faces a crisis from viral infections, effective vaccines contribute to protection against the virus [25]. Several vaccines have been developed against SARS-CoV-2 and, as of 18 May 2021, more than one billion COVID-19 vaccine doses have been administered worldwide [1]. Based on the results from clinical trials, the FDA approved three types of COVID-19 vaccine: Pfizer-BioNTech/BNT162b2 (mRNA) [26], Moderna/mRNA 1273 (mRNA) [27], and Janssen/Ad26.COV 2.S (viral vector) [28]. Meanwhile, the Korean Ministry of Food and Drug Safety approved Pfizer-BioNTech/BNT162b2 [26] and AstraZeneca/AZD1222 (viral vector) COVID-19 vaccines [29] in February, 2021. However, the drive for COVID-19 vaccination is slow in Korea: only 2.1 million people were completely vaccinated in the last three months (March–May) [30].

Cancer patients’ willingness to take the COVID-19 vaccination might be affected by many factors, such as individuals’ belief in vaccination, clinicians’ recommendations, and socioeconomic status. The French and Polish survey studies commonly identified male patients, older age, and history of influenza vaccination as positive predictors for the acceptance of COVID-19 vaccination [20,21], consistent with our study. These three predictors were also reported as predictors for influenza and COVID-19 vaccination in general populations [31,32]. Furthermore, the Polish survey study reported that education, marital status, and active cancer treatment were significantly associated with willingness to take the COVID-19 vaccination [21], but none of these three factors affected patients’ willingness in our study. Such inconsistent results might originate from the different study populations: besides the geographic and cultural differences, the distribution of primary cancer types and proportion of patients under active treatment at the time of the survey also differed (47%, 61.9%, and 73.3% in the French, Polish, and our study, respectively) [20,21].

In this study, we also found that overweight to obese cancer patients showed an increased acceptance rate of the COVID-19 vaccination, which might reflect the well-known report that people with obesity are at an increased risk of severe COVID-19 and higher mortality [33]. Notably, while the primary site of malignancy did not affect cancer patients’ acceptance rate of COVID-19 vaccination, cancer patients who did not experience disease recurrence and who survived ≥5 years from initial cancer diagnosis tended to accept the COVID-19 vaccination. Moreover, EQ-5D-3L health score and VAS were positively associated with the acceptance rate of COVID-19 vaccination. This study is the first to report that cancer patients’ wellness or perception of being of good health status could positively influence their decision to take the COVID-19 vaccination.

The most common reason for not accepting COVID-19 vaccination was concerns about the COVID-19 vaccine’s safety (161/385 respondents, 41.8%). In particular, approximately 60% of those who experienced cancer recurrence, those who have not yet reached five years post initial cancer diagnosis, and those with lower VAS scores expressed concerns about vaccine safety, revealing that they were more sensitive to the safety issue and were trying to avoid the associated uncertainty. Regarding the COVID-19 vaccine efficacy and safety, especially for cancer patients, convincing and robust results have not yet been reported. However, it is evident that the benefits of taking the COVID-19 vaccine outweigh the risks, considering the severe detrimental effects of COVID-19 in patients with underlying malignancies [34]. Cancer patients who have serious health conditions are more vulnerable than patients in better conditions; therefore, they are the very population who urgently require the COVID-19 vaccine.

Impressively, in this survey, 91.2% of cancer patients were willing to be vaccinated if their attending physicians recommend it, indicating that almost 30% could change their decision upon their physicians’ recommendation. Unlike the other factors, which are unmodifiable, physician recommendation is the single modifiable factor that could change patients’ behaviour.

Our study has several limitations. First, although we included approximately a thousand Korean cancer patients, participation bias might exist, affecting the outcomes. Cancer patients’ expectations of vaccine efficacy or anxiety about vaccine side effects, which might affect their participation in the survey and the acceptance rate of COVID-19 vaccination, were not investigated. Second, the distribution of cancer types in this study differed from the current cancer statistics in Korea [35]. Third, there were more female respondents in this study than male. It is known that women are more likely to participate in surveys than men, which necessitates statistical correction in our survey [36]. Fourth, we excluded the healthy adult population; therefore, results from the study population could not be compared with those from the control group. Nevertheless, to our knowledge, this is the first study exploring Korean cancer patients’ acceptance rate of being vaccinated against COVID-19 at the national level. The factors that were possibly related to such attitudes were also investigated.

## 5. Conclusions

In conclusion, our results demonstrate that 61.8% of cancer patients were willing to receive the COVID-19 vaccination. Along with the previously reported predictors for COVID-19 vaccination, including male gender, older age, and influenza vaccination history, we found that cancer patients’ disease status and current health status were associated with acceptance of COVID-19 vaccination. Considering that COVID-19 vaccination hesitancy among cancer patients could be significantly averted by physician recommendations, physicians should play a major role in aiding cancer patients’ decision-making concerning the COVID-19 vaccination.

## Figures and Tables

**Table 1:** Patients’ characteristics.

Characteristics	All (*n* = 1001, %)	Intention to COVID-19 Vaccination			
		Yes (*n* = 608, %)	Unsure (*n* = 277, %)	No (*n* = 108, %)	*p*-Value
Age, years					
Mean ± SD	57.4 ± 12.0	59.0 ± 11.6	54.5 ± 12.0	56.3 ± 12.8	<0.001
<50	265 (26.5)	129 (21.2)	98 (35.4)	35 (32.4)	<0.001
≥50	736 (73.5)	479 (78.8)	179 (64.6)	73 (67.6)	
Sex					<0.001
Male	283 (28.3)	216 (35.5)	48 (17.3)	18 (16.7)	
Female	718 (71.7)	392 (64.5)	229 (82.7)	90 (83.3)	
BMI ^a^, kg/m^2^					
Mean ± SD	23.3 ± 3.8	23.6 ± 3.9	23.0 ± 3.7	22.9 ± 3.7	0.027
<23.0 (underweight to normal)	483 (48.3)	268 (44.2)	147 (53.1)	64 (59.3)	0.003
≥23.0 (overweight to obesity)	517 (51.7)	339 (55.8)	130 (46.9)	44 (40.7)	
Occupation ^b^					0.113
Yes	270 (27.0)	179 (29.4)	66 (23.8)	24 (22.4)	
No	730 (73.0)	429 (70.6)	211 (76.2)	83 (77.6)	
Education ^c^					0.280
≤High school	626 (62.7)	391 (64.4)	168 (60.6)	62 (57.4)	
≥College	373 (37.3)	216 (35.6)	109 (39.4)	46 (42.6)	
Marital status ^d^					0.274
Single/divorced/bereavement	221 (22.1)	125 (20.6)	70 (25.4)	23 (21.3)	
Married/living together	778 (77.9)	483(79.4)	206 (74.6)	85(78.7)	
No. of people living with ^e^					0.495
≤2	727 (72.8)	449 (74.0)	194 (70.3)	77 (71.3)	
>2	272 (27.2)	158 (26.0)	82 (29.7)	31 (28.7)	
Household income ^f^, USD/month					0.442
≤1800	302 (30.4)	192 (31.6)	78 (28.6)	29 (27.4)	
1800–3600	307 (30.9)	175 (28.8)	94 (34.4)	37 (34.9)	
>3600	384 (38.7)	240 (39.5)	101 (37.0)	40 (37.7)	
Residence ^g^					0.105
Seoul metropolitan area	617 (61.8)	389 (64.0)	169 (61.5)	57 (53.3)	
Other area	381 (38.2)	219 (36.0)	106 (38.5)	50 (46.7)	
Smoking ^h^					0.001
Never	681 (68.4)	384 (63.7)	213 (76.9)	79 (73.2)	
Ever	271 (27.3)	186 (30.8)	59 (21.3)	24 (22.2)	
Current	43 (4.3)	33 (5.5)	5 (1.8)	5 (4.6)	
Comorbidity ^i^					
Hypertension	241 (24.1)	166 (27.3)	50 (18.1)	24 (22.2)	0.010
Diabetes	102 (10.2)	70 (11.5)	23 (8.3)	9 (8.3)	0.270
Dyslipidemia	107 (10.7)	64 (10.5)	32 (11.6)	11 (10.2)	0.882
Cardiovascular disease	22 (2.2)	18 (3.0)	4 (1.4)	0	0.092
Bone/joint disease	53 (5.3)	28 (4.6)	17 (6.1)	7 (6.5)	0.528
Pulmonary/allergic disease	28 (2.8)	17 (2.8)	7 (2.5)	4 (3.7)	0.821
No. of comorbidity ^j^					0.279
0	532 (54.2)	316 (53.2)	157 (57.3)	54 (50.9)	
1	280 (28.5)	164 (27.6)	78 (28.5)	36 (34.0)	
≥2	169 (17.2)	114 (19.2)	39 (14.2)	16 (15.1)	
Primary site of cancer ^k^					0.260
1	894 (89.4)	535 (88.0)	251 (90.6)	101 (93.5)	
2	86 (8.6)	57 (9.4)	22 (7.9)	7 (6.5)	
≥3	20 (2.0)	16 (2.6)	4 (1.5)	0	

Abbreviations. BMI, body mass index; COVID-19, coronavirus disease 2019; SD, standard deviation. Missing data. ^a^ 1; ^b^ 1; ^c^ 2; ^d^ 2; ^e^ 2; ^f^ 8; ^g^ 3; ^h^ 6; ^I^ 20; ^j^ 20; ^k^ 1.

**Table 2:** Cancer diagnosis and treatment.

Characteristics	All (*n* = 1001, %)	Intention to COVID-19 Vaccination			
		Yes (*n* = 608, %)	Unsure (*n* = 277, %)	No (*n* = 108, %)	*p*-Value
Primary site of cancer ^a^					
Lung	91 (91)	59 (9.7)	24 (8.7)	8 (7.4)	0.706
Stomach	102 (10.2)	81 (13.3)	14 (5.1)	6 (5.6)	<0.001
Colorectum	138 (13.8)	87 (14.3)	36 (13.0)	13 (12.0)	0.756
Prostate	42 (4.2)	33 (5.4)	7 (2.5)	2 (1.9)	0.060
Liver	24 (2.4)	17 (2.8)	5 (1.8)	2 (1.9)	0.620
Thyroid	58 (5.8)	34 (5.6)	22 (7.9)	2 (1.9)	0.067
Pancreas	5 (0.5)	1 (0.2)	1 (0.4)	3 (2.8)	0.013
Gallbaldder/bile duct	6 (0.6)	6 (1.0)	0	0	0.237
Kidney	16 (1.6)	13 (2.1)	3 (1.1)	0	0.259
Bladder	13 (1.3)	10 (1.6)	2 (0.7)	1 (0.9)	0.645
Leukemia/lymphoma	26 (2.6)	19 (3.1)	4 (1.4)	3 (2.8)	0.346
Breast	209 (20.9)	119 (19.6)	56 (20.2)	31 (28.7)	0.095
Uterine cervix	74 (7.4)	45 (7.4)	19 (6.9)	10 (9.3)	0.721
Uterine corpus	65 (6.5)	38 (6.3)	21 (7.6)	6 (5.6)	0.689
Ovary	202 (20.2)	104 (17.1)	73 (26.4)	24 (22.2)	0.006
Others	59 (5.9)	34 (5.6)	21 (7.6)	4 (3.7)	0.296
Treatment of cancer ^b^					
Chemotherapy	681 (68.0)	388 (63.8)	206 (74.4)	80 (74.1)	0.003
Surgery	665 (66.4)	398 (65.5)	193 (69.7)	68 (63.0)	0.533
Radiation	268 (26.8)	158 (26.0)	76 (27.4)	31 (28.7)	0.044
Targeted agent	228 (22.8)	131 (21.5)	61 (22.0)	35 (32.4)	0.043
Hormone therapy	109 (10.9)	58 (9.5)	29 (10.5)	19 (17.6)	0.188
Immunotherapy	99 (9.9)	60 (9.9)	31 (11.2)	8 (7.4)	0.343
Endoscopic resection	57 (5.7)	38 (6.3)	16 (5.8)	2 (1.9)	0.796
Others	27 (2.7)	18 (3.0)	6 (2.2)	3 (2.8)	0.796
No. of treatment modality ^c^					0.225
Single	346 (34.8)	223 (37.1)	89 (32.1)	33 (30.8)	
Multiple	647 (65.2)	378 (62.9)	188 (67.9)	74 (69.2)	
Time from initial cancer diagnosis ^d^, years					0.120
<5	796 (79.9)	472 (77.9)	227 (82.2)	91 (85.0)	
≥5	200 (20.1)	134 (22.1)	49 (17.8)	16 (15.0)	
Currently on treatment ^e^					0.764
Yes	731 (73.3)	438 (72.3)	206 (74.6)	79 (73.1)	
No	267 (26.8)	168 (27.7)	70 (25.4)	29 (26.9)	
Recurrence ^f^					0.207
Yes	267 (26.8)	151 (24.9)	84 (30.3)	31 (29.0)	
No	731 (73.3)	456 (75.1)	193 (69.7)	76 (71.0)	

Abbreviation. COVID-19, coronavirus disease 2019. Missing data. ^a^ 1; ^b^ 8; ^c^ 8; ^d^ 5; ^e^ 3; ^f^ 3.

**Table 3:** Respondents’ reasons for accepting or not accepting COVID-19 vaccination.

Acceptance Group (*n* = 608, %)	
Trust in COVID-19 vaccine efficacy	284 (46.7)
Because of an underlying malignancy	185 (30.4)
Trust in COVID-19 vaccine safety	69 (11.4)
Recommendation from health authorities	39 (6.4)
Recommendation from press	18 (3.0)
Others	13 (2.1)
**No Acceptance Group (*n* = 385, %)**	
Concerns about COVID-19 vaccine safety	161 (41.8)
Mistrust in COVID-19 vaccine efficacy	79 (20.5)
Lack of information about COVID-19 vaccine	59 (15.3)
Because of an underlying malignancy	42 (10.9)
Mistrust of vaccination itself	13 (3.4)
Others	31 (8.1)

Abbreviation. COVID-19, coronavirus disease 2019.

**Table 4:** Results of univariable analyses for COVID-19 vaccination acceptance.

Variables	N (Acceptance)	Univariable Analysis		Stratified Univaraibe Analysis	
		OR (95% CI)	*p*-value	OR (95% CI)	*p*-Value
Sex					
Male	282 (216)	1			
Female	711 (392)	0.38 (0.28–0.51)	<0.001		
Age, years					
<50	262 (129)	1		1	
≥50	731 (479)	1.96 (1.47–2.61)	<0.001	1.68 (1.25–2.25)	0.001
BMI, kg/m^2^					
<23.0 (underweight to normal)	479 (268)	1		1	
≥23.0 (overweight to obesity)	513 (339)	1.53 (1.19–1.98)	0.001	1.42 (1.10–1.85)	0.008
Currently on treatment					
No	267 (168)	1		1	
Yes	723 (438)	0.91 (0.68–1.21)	0.503	0.83 (0.62–1.12)	0.219
Recurrence					
No	725 (456)	1		1	
Yes	266 (151)	0.78 (0.58–1.03)	0.080	0.77 (0.57–1.03)	0.076
Residence					
Other area	375 (219)	1		1	
Seoul metropolitan area	615 (389)	1.23 (0.94–1.60)	0.128	1.17 (0.89–1.52)	0.264
Smoking					
Never	676 (384)	1		1	
Ever	269 (186)	1.70 (1.26–2.30)	0.001	0.86 (0.57–1.29)	0.464
Current	43 (33)	2.51 (1.22–5.17)	0.013	1.33 (0.61–2.89)	0.474
Influenza vaccination within 3 years					
No	245 (125)	1		1	
Yes	740 (480)	1.77 (1.32–2.37)	<0.001	1.85 (1.37–2.49)	<0.001
No. of comorbidity					
0	527 (316)	1		1	
1	278 (164)	0.96 (0.72–1.29)	0.790	0.97 (0.72–1.31)	0.852
≥2	169 (114)	1.38 (0.96–2.00)	0.082	1.29 (0.89–1.88)	0.176
Education					
≤High school	621 (391)	1		1	
≥College	371 (216)	0.82 (0.63–1.07)	0.138	0.83 (0.63–1.08)	0.168
Immunotherapy					
No	886 (541)	1		1	
Yes	99 (60)	0.98 (0.64–1.50)	0.930	0.96 (0.62–1.49)	0.860
No. of treatment modality					
Single	345 (223)	1		1	
Multiple	640 (378)	0.79 (0.60–1.04)	0.087	0.91 (0.69–1.21)	0.511
Time from initial cancer diagnosis, years					
<5	790 (472)	1		1	
≥5	199 (134)	1.39 (1.00–1.93)	0.050	1.47 (1.05–2.05)	0.024
EQ-5D-3L health score, 0.1-point increase	991 (607)	1.14 (1.02–1.27)	0.018	1.12 (1.01–1.26)	0.041
VAS, 10-point increase	989 (607)	1.12 (1.04–1.21)	0.002	1.12 (1.04–1.21)	0.003

Abbreviations: aOR, adjusted odds ratio; BMI, body mass index; CI, confidence interval; COVID-19, corona virus disease 2019; EQ-5D-3L, EuroQol-5 Dimension 3-level questionnaire; OR, odds ratio; SD, standard deviation; VAS, visual analogue scale.

## Data Availability

External researchers can make written requests to the corresponding author (M.C.L.) for sharing of data before publication or presentation. Requests with submitting a brief analysis plan and synopsis will be assessed and approved on a case-by-case basis in the research team. The personal identification deleted data will be sent in password-protected files. All data sharing will abide by rules and policies; relevant institutional review boards; and local, state, and federal laws and regulations. Data sharing mechanisms will ensure that the rights and privacy of individuals participating in research will be guaranteed.

## Supplementary Materials

The following are available online at https://www.mdpi.com/article/10.3390/cancers13153883/s1, Supplementary Material. English version of survey questionnaire. Table S1. Results of the EQ-5D-3L; Table S2. Personal experiences during COVID-19 pandemic.
