# Supplementary material for: Cancer Patients’ Willingness to Take COVID-19 Vaccination: A Nationwide Multicenter Survey in Korea
Source: Cancers (Basel). 2021 Aug 1;13(15):3883. doi: 10.3390/cancers13153883 (PMC8345425; doi:10.3390/cancers13153883)
Supplement: Supplementary file 1 [file cancers-13-03883-s001.zip › Supplementary Material (Questionnaire English).pdf]

Version 1.0 (2021-01-12)

ID	-			-				

Questionnaire on experiences and thoughts related to COVID-19 and vaccines

Greetings!

The recent COVID-19 pandemic has had a substantial impact on our lives. This survey intends to examine cancer patients' experiences and thoughts about COVID-19 and the COVID-19 vaccines.

There are no right or wrong answers. Your responses will only be used for statistical purposes, so please answer the questions as you normally would. In addition, your personal information will be strictly protected under the Personal Information Protection Act. There will be a follow-up survey approximately one year after this one.

Please provide your consent to participate in the study, and then complete the questionnaire. Thank you for your time.

January 2021

Managing organization	국립암센터 NATIONAL CANCER CENTER
Participating institutions	Seoul National University Hospital,<br>Jeju National University Hospital,<br>Chungnam National University Sejong Hospital<br>Ulsan University Hospital

1. I have understood the contents of this survey and am voluntarily participating in this study.

□ I agree. □ I disagree.

2. I give my permission to collect and provide my contact information to the survey organization for a follow-up on whether I have received the COVID-19 vaccination.

□ Yes, I do. Phone number__________________ □ No, I do not.

Purpose	Follow-up survey on whether COVID-19 vaccination was received		
Collected personal information	Phone number and name		
Retention period and disposal	Discarded immediately after the follow-up survey	Agency receiving the information	Follow-up survey agency

Date:

Participant:__________________(Signature)

Researcher:__________________(Signature)

We will ask you a few questions about your experiences with COVID-19.

Q1) Have you personally experienced the following in relation to COVID-19?

Item		Yes	No
1	I have been tested for COVID-19.	①	②
2	I have been diagnosed (confirmed) with COVID-19.	①	②
3	I have been under self-quarantine due to close contact with a patient.	①	②
4	I have been under self-quarantine after entry into Korea from a foreign country.	①	②
5	People I know (friends, family, relatives, coworkers, etc.) have been diagnosed with COVID-19.	①	②
6	People I know (friends, family, relatives, coworkers, etc.) have self-quarantined.	①	②

Q2) Have you personally experienced the following in terms of health care during the COVID-19 pandemic?

Item		Yes	No
1	I have postponed my outpatient appointment.	①	②
2	My hospitalization has been postponed.	①	②
3	I could not receive treatment/had changes to planned treatment.	①	②
4	I could not undergo testing/had changes to planned testing.	①	②

Q3) How well have you complied with the following practices during the COVID-19 pandemic?

Item		Always	Mostly	Moderately	Rarely	Never
1	Wearing a face mask	①	②	③	④	⑤
2	Washing hands for at least 30 seconds	①	②	③	④	⑤
3	Using an alcohol hand sanitizer	①	②	③	④	⑤
4	Social distancing	①	②	③	④	⑤
5	Refraining from attending personal gatherings (gatherings with family/friends, religious activities, etc.)	①	②	③	④	⑤

We will ask you a few questions on your thoughts about COVID-19 and the COVID-19 vaccines.

Q4) How would you predict your likelihood of contracting COVID-19 in the next 6 months?

① I will not be infected
② I may be infected but will be asymptomatic
③ I may be infected but have mild symptoms
④ I may be infected and have severe symptoms

Q5) If COVID-19 vaccines are introduced, are you willing to get vaccinated?

① Yes → **Q6-1)**   ② No → **Q6-2)**   ③ I do not know → **Q6-2)**

Q6-1) (If yes on Q5) Choose **one primary** reason that you want to get vaccinated.

① I trust the efficacy of the COVID-19 vaccine
② I believe that the COVID-19 vaccine will have minimal side effects
③ Due to the positive news reports
④ Due to the positive announcements by the Korea Disease Control and Prevention Agency
⑤ I have cancer/chronic diseases
⑥ I want to travel within and outside the country
⑦ It is recommended by family, friends, and others
⑧ Other (   )

Q6-2) (If no or I do not know on Q5) Choose **one primary** reason that you do not want to get vaccinated.

① I do not trust the efficacy of the COVID-19 vaccine
② I believe that the COVID-19 vaccine will have severe side effects
③ I think vaccination is unnecessary
④ I do not have time to get vaccinated
⑤ Due to the negative media coverage
⑥ The Korea Disease Control and Prevention Agency did not give a definitive statement
⑦ Due to lack of information about the types and efficacy/side effects of the vaccine
⑧ It is not recommended by family, friends, and others
⑨ Other ( )

Q7) Which of the following influences your decision regarding COVID-19 vaccination? Please choose **the (one) greatest influence.**

① Press and public media reports
② Opinions of the Ministry of Health and Welfare and the Korea Disease Control and Prevention Agency
③ Recommendations by my doctor/oncologist
④ Opinions of family, friends, and others
⑤ Other ( )

Q8) **If your doctor or oncology specialist** recommends that you receive the COVID-19 vaccine, will you get the vaccine?

① Yes ② No

Q9) If you **receive** the COVID-19 vaccine, what do you think are the **odds of you getting infected by COVID-19** in the next 6 months?

① I will not be infected
② I may be infected but will be asymptomatic
③ I may be infected but have mild symptoms
④ I may be infected and have severe symptoms.

Q10) Please check the boxes that best represent **your thoughts** regarding COVID-19.

Item		Yes	No
1	If infected with COVID-19, cancer patients are more likely to develop severe complications than the general population	①	②
2	If infected with COVID-19, cancer patients are more likely to die than the general population	①	②
3	Cancer patients are prioritized over other patients to receive the COVID-19 vaccine	①	②

We will ask you some questions related to vaccinations, such as flu (influenza) vaccines.

Q11) Have you received the flu (influenza) vaccine since September 2020?

① Yes → **Q12)** ② No→ **Q11-1)**

Q11-1) If you have not received the flu vaccine since September 2020, what is the reason? Please choose **the (one) greatest reason**.

①I do not trust the efficacy of the flu vaccine

② I believe that the flu vaccine will have severe side effects

③ I think vaccination is unnecessary

④ I do not have time to get vaccinated

⑤ Due to the negative media coverage

⑥ My doctor/oncology specialist told me not to

⑦ I had side effects from a vaccine in the past

⑧ It's too expensive

⑨ Other ( )

Q12) How many times have you received the flu vaccine **in the past 3 years** (since Fall 2017)?"

① 0 ② 1 ③ 2 ④ 3

Q13) What was the minimum age for free flu vaccines at public health centers after September 2020?

① 61 ② 62 ③ 63 ④ 64 ⑤ 65

Q14) Have you ever received the **pneumococcal vaccine?** If so, what type of vaccine did you get?

① No ② Yes (□ 13-valent □ 23-valent □ I do not know the type)

Q15) Have you ever experienced **side effects** after getting a vaccine?

① Yes → **Q15-1)** ② No → **Q16)**

Q15-1) To which **vaccine(s)** did you develop **adverse reactions**? Mark **all** of the vaccines.

□Flu □Pneumococcal □Shingles □Diphtheria/Tetanus/Pertussis

□Hepatitis A □ Hepatitis B □Measles/Mumps/Rubella □Other (____________)

Q15-2) What type of **adverse reactions** to **vaccines** did you experience? Please **mark all of them**.

① Adverse reaction at the injection site:

□Pain □Skin rash □Swelling □Itching □Other (____________)

② Systemic adverse reaction:

□Fever □Allergic reaction □Headache □Fatigue □Muscle ache

□Nausea □Vomiting □Diarrhea □Other (____________)

Q15-3) How **severe** was the **most adverse** reaction you experienced?

① Life-threatening (went to the ER)

② Mild enough to not interfere with daily life

③ Serious enough to interfere with daily life (took medications or got a shot)

Cancer diagnosis and treatment

Q16) Have you ever been **diagnosed with cancer?**

① Yes → **Q16-1)** ② No → **Q17)**

Q16-1) Which cancer(s) were you diagnosed with? Please **mark all of them**.

□Lung cancer □Gastric cancer □Colorectal cancer □Prostate cancer

□Liver cancer □Thyroid cancer □Pancreatic cancer

□Gallbladder cancer/cholangiocarcinoma □Kidney cancer □Urinary bladder cancer

□Leukemia/Lymphoma □Breast cancer □Cervical cancer □ Endometrial cancer
□Ovarian cancer □Other (____________)

Q16-2) **When were you diagnosed with cancer?** If you have been diagnosed with multiple cancers, please write the date of the diagnosis of the first cancer. If you do not remember the date of diagnosis, please specify the first day of treatment.

( ) Year ( ) Month

(Example: Date of surgery or first day of chemotherapy)

Q16-3) Are you currently **being treated for cancer**?

① Yes ② No

Q16-4) Which type of cancer treatment have you received? Please **mark all of them**.

□Chemotherapy □Immunotherapy □Hormone therapy □ Target therapy
□Procedure (Endoscopy, etc.) □Surgery □Radiation therapy
□Other (____________)

Q16-5) Have you ever had a **cancer relapse?**

① Yes ② No

Q17) Have you been diagnosed with or treated for any of the following diseases in the past year? If so, please **mark all of them**.

□None □Hypertension □Dyslipidemia (hyperlipidemia) □Stroke
□Myocardial infarction □ Angina □Osteoarthritis □Rheumatoid arthritis
□Osteoporosis □Pulmonary tuberculosis □Asthma □Diabetes mellitus
□Thyroid disease □Depression □Atopic dermatitis □Allergic rhinitis
□Sinusitis □Otitis media □Cataract □Glaucoma □Macular degeneration
□Renal failure □Hepatitis B □Hepatitis C □Liver cirrhosis □Other(________________)

Q18) Are you currently a **smoker**?

① Yes
② I was a smoker in the past
③ I have never been a smoker (consumed less than 5 packs of cigarettes in my lifetime)

Today’s health status

※ Under each heading, please check the ONE box that best describes your health TODAY.

Q19) Mobility

① I have no problems in walking about

② I have some problems in walking about

③ I am confined to bed

Q20) Self-care

① I have no problems with self-care

② I have some problems washing or dressing myself

③ I am unable to wash or dress myself

Q21) Usual activities (e.g., work, study, housework, family or leisure activities)

① I have no problems with performing my usual activities

② I have some problems with performing my usual activities

③ I am unable to perform my usual activities

Q22) Pain/discomfort

① I have no pain or discomfort

② I have moderate pain or discomfort

③ I have extreme pain or discomfort

Q23) Anxiety/depression

① I am not anxious or depressed

② I am moderately anxious or depressed

③ I am extremely anxious or depressed

- We would like to know how good or bad your health is TODAY.
- This scale is numbered from 0 to 100.
- 100 means the best health you can imagine.

  0 means the worst health you can imagine.
- Mark an X on the scale to indicate how your health is TODAY.
- Now, please write the number you marked on the scale in the box below.

OUR HEALTH TODAY = [ ]

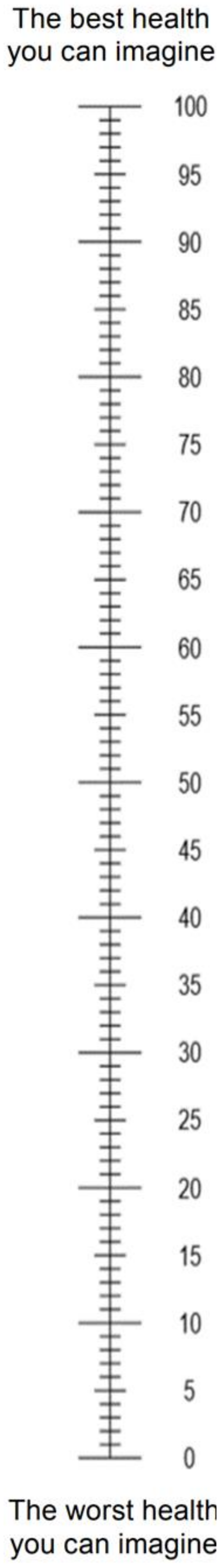

Demographic information

※ Please fill out the following information.

Age	___________ years	Sex	① Male ② Female
Height	________________cm	Weight	______ kg
Employment status	① Employed<br>② On leave<br>③ Unemployed (housewife, student, etc.)		
Highest education completed	① Middle school or lower<br>② High school<br>③ College<br>④ Graduate school or higher		
Marital status	① Single<br>② Married<br>③ Not married but living together<br>④ Divorced/separated<br>⑤ Widowed		
Number of cohabitants	How many people do you live with? (excluding yourself) _______		
Average monthly household income	Please specify your average monthly household income including the national basic livelihood subsidy.<br>※ Combining the income of all members of the family (rental income, pension)<br>① 2 million KRW or lower<br>② More than 2 million KRW to less than or equal to 4 million KRW<br>③ More than 4 million KRW to less than or equal to 6 million KRW<br>④ More than 6 million KRW to less than or equal to 8 million KRW<br>⑤ More than 8 million KRW		
Current area of residence	__________Province (Metropolitan city) ___________city (gun, gu)		
