# Supplementary material for: Cancer Patients’ Willingness to Take COVID-19 Vaccination: A Nationwide Multicenter Survey in Korea
Source: Cancers (Basel). 2021 Aug 1;13(15):3883. doi: 10.3390/cancers13153883 (PMC8345425; doi:10.3390/cancers13153883)
Supplement: Supplementary file 1 [file cancers-13-03883-s001.zip › Table S1.pdf]

Table S1. Results of the EQ-5D-3L

Characteristics	All (*n* = 1001, %)	Intention to COVID-19 vaccination			
		Yes (*n* = 608, %)	Unsure (*n* = 277, %)	No (*n* = 108, %)	*p*-value
Mobility[a]					0.755
No problems	777 (77.8)	475 (78.3)	215 (77.6)	81 (75.0)	
Other answers	222 (22.2)	132 (21.7)	62 (22.4)	27 (25.0)	
Self-care[b]					0.566
No problems	911 (91.3)	558 (91.9)	251 (90.9)	96 (88.9)	
Other answers	87 (8.7)	49 (8.1)	25 (9.1)	12 (11.1)	
Usual activities[c]					0.370
No problems	772 (77.2)	478 (78.6)	208 (75.1)	80 (74.1)	
Other answers	228 (22.8)	130 (21.4)	69 (24.9)	28 (25.9)	
Pain/discomfort[d]					0.023
No problems	540 (54.0)	348 (57.2)	131 (47.3)	58 (53.7)	
Other answers	460 (46.0)	260 (42.8)	146 (52.7)	50 (46.3)	
Anxiety/depression[e]					0.005
No problems	623 (62.4)	400 (65.9)	151 (54.5)	69 (63.9)	
Other answers	376 (37.6)	207 (34.1)	126 (45.5)	39 (36.1)	
EQ-5D-3L health score[f]					
Mean ± SD	0.9±0.1	0.9±0.1	0.9±0.1	0.9±0.1	0.038
Median (range)	0.9 (-0.01–1)	0.9 (0.3–1)	0.9 (0.4–1)	0.9 (0.5–1)	0.020
VAS[g]					
Mean ± SD	72.2±17.7	73.6±17.3	70.4±18.4	69.1±16.0	0.008
Median (range)	75 (10–100)	80 (10–100)	70 (10–100)	70 (30–95)	0.004

Abbreviations. COVID-19, corona virus disease 2019; EQ-5D-3L, EuroQol-5 Dimension 3-level questionnaire; SD, standard deviation; VAS, visual analogue scale.

Missing data. [a]2; [b]3; [c]1; [d]1; [e]2; [f]3; [g]5.
