# Supplementary material for: Cancer Patients’ Willingness to Take COVID-19 Vaccination: A Nationwide Multicenter Survey in Korea
Source: Cancers (Basel). 2021 Aug 1;13(15):3883. doi: 10.3390/cancers13153883 (PMC8345425; doi:10.3390/cancers13153883)
Supplement: Supplementary file 1 [file cancers-13-03883-s001.zip › Table S2.pdf]

Table S2. Personal experiences during COVID-19 pandemic

Characteristics	All (*n* = 1001, %)	Intention to COVID-19 vaccination			
		Yes (*n* = 608, %)	Unsure (*n* = 277, %)	No (*n* = 108, %)	*p*-value
COVID-19 test	557 (55.6)	326 (53.6)	164 (59.2)	62 (57.4)	0.277
COVID-19 diagnosis	3 (0.3)	2 (0.3)	1 (0.4)	0	>0.999
Self-isolation as a close contact	13 (1.3)	6 (1.0)	6 (2.2)	1 (0.9)	0.364
There is a person diagnosed with COVID-19 nearby (friends, relatives, co-workers, etc.)	40 (4.0)	27 (4.4)	9 (3.3)	4 (3.7)	0.694
There is a person who had self-isolation as a close contact nearby (friends, relatives, co-workers, etc.)	106 (10.6)	62 (10.2)	30 (10.8)	13 (12.0)	0.837
Delayed scheduled outpatient clinic visit	43 (4.3)	22 (3.6)	13 (4.7)	8 (7.4)	0.192
Delayed scheduled hospital admission[a]	28 (2.8)	13 (2.1)	8 (2.9)	6 (5.6)	0.129
Not received and/or changed planned treatment[b]	26 (2.6)	15 (2.5)	8 (2.9)	3 (2.8)	0.932
Not received and/or changed planned test	24 (2.4)	14 (2.3)	7 (2.5)	3 (2.8)	0.948

Abbreviation. COVID-19, corona virus disease 2019.
Missing data. [a]1; [b]1.
